# Pretransplant characteristics of kidney transplant recipients that predict posttransplant outcome

**DOI:** 10.3389/fimmu.2022.945288

**Published:** 2022-07-25

**Authors:** Martin Tepel, Subagini Nagarajah, Qais Saleh, Olivier Thaunat, Stephan J. L. Bakker, Jacob van den Born, Morten A. Karsdal, Federica Genovese, Daniel G. K. Rasmussen

**Affiliations:** ^1^ Department of Nephrology, Odense University Hospital, Odense, Denmark, and Cardiovascular and Renal Research, Institute of Molecular Medicine, Clinical Institute, University of Southern Denmark, Odense, Denmark; ^2^ Hospices Civils de Lyon, Hôpital Edouard Herriot, Service de Transplantation, Néphrologie et Immunologie Clinique, Lyon, France; ^3^ Division of Nephrology, Department of Medicine, University Medical Center Groningen, University of Groningen, Groningen, Netherlands; ^4^ Nordic Bioscience, Herlev, Denmark

**Keywords:** kidney transplantation, potential kidney transplant recipient, biomarker, immunology, precision transplant medicine, endotrophin, rejection, acute allograft injury

## Abstract

Better characterization of the potential kidney transplant recipient using novel biomarkers, for example, pretransplant plasma endotrophin, will lead to improved outcome after transplantation. This mini-review will focus on current knowledge about pretransplant recipients’ characteristics, biomarkers, and immunology. Clinical characteristics of recipients including age, obesity, blood pressure, comorbidities, and estimated survival scores have been introduced for prediction of recipient and allograft survival. The pretransplant immunologic risk assessment include histocompatibility leukocyte antigens (HLAs), anti-HLA donor-specific antibodies, HLA-DQ mismatch, and non-HLA antibodies. Recently, there has been the hope that pretransplant determination of markers can further improve the prediction of posttransplant complications, both short-term and long-term outcomes including rejections, allograft loss, and mortality. Higher pretransplant plasma endotrophin levels were independently associated with posttransplant acute allograft injury in three prospective European cohorts. Elevated numbers of non-synonymous single-nucleotide polymorphism mismatch have been associated with increased allograft loss in a multivariable analysis. It is concluded that there is a need for integration of clinical characteristics and novel molecular and immunological markers to improve future transplant medicine to reach better diagnostic decisions tailored to the individual patient.

## Introduction

In patients with end-stage kidney disease, kidney transplantation can improve their health and quality-adjusted life years (1). However, kidney transplant function may be unsatisfactory in some kidney transplant recipients because of acute allograft injury after transplant, episodes of rejections, or infections (2). According to the United Network for Organ Sharing (UNOS) database, approximately 12% of transplant recipients are waiting for a second transplant (3), which may be due to several causes including incomplete characterization of the potential kidney transplant recipient (3, 4).

Apparently, it still is difficult to predetermine allograft failure for an individual kidney transplant recipient. Studies indicated that the estimated glomerular filtration rate (5, 6) or proteinuria (7) may not predict future allograft failure. To improve the prediction of allograft failure, Foucher et al. reported the Kidney Transplant Failure Score including recipient age, gender, creatinine, previous transplants, donor creatinine, recipient serum creatinine 3 months posttransplant and 12 months posttransplant, rejection episodes, and proteinuria 12 months posttransplant (8). However, this score largely depends on long-term follow-up of kidney function for outcome prediction. Loupy et al. reported the iBox score (9), showing that time of posttransplant risk evaluation, estimated glomerular filtration rate, proteinuria, allograft histology including interstitial fibrosis, tubular atrophy, glomerulitis, peritubular capillaritis, interstitial inflammation, tubulitis, allograft glomerulopathy, and anti-HLA donor-specific antibodies were all independent predictors of long-term allograft failure in a multivariable analysis (9). However, the score depends on several posttransplant data and there still is the need for an invasive kidney allograft biopsy.

One step toward improved outcome and better decisions tailored to the individual patient may be the use of novel markers and characteristics (10–13). On the one hand, potential markers influencing posttransplant outcome may show several interrelationships. On the other hand, the allocation of donor kidneys should result in maximum benefit for potential recipients (14). The present review will focus on characteristics and markers which are available before transplantation for prediction of posttransplant outcome, complications, and survival of patients and allografts.

## Clinical characteristics and posttransplant outcome

Clinical characteristics in potential kidney transplant recipients which can be obtained before transplant and may affect kidney transplant outcome include age, dialysis modality, obesity, blood pressure, and comorbidities. Intuitively, one can understand that recipients’ age and pretransplant dialysis modality may affect transplant outcome. Data of 189,141 first adult kidney transplant recipients from the United Network for Organ Sharing (UNOS) database indicated that recipient age affected allograft survival (15). The increased recipient age also predicted increased patient mortality (16). A retrospective analysis showed that pretransplant hemodialysis patients had a lower estimated glomerular filtration rate 1-year posttransplant compared to pretransplant peritoneal dialysis patients (17). Furthermore, pretransplant hemodialysis patients had higher risks of death-censored graft failure compared to pretransplant peritoneal dialysis patients (18).

Obesity may be important, i.e., a body mass index above 30 kg/m^2^ is known to be associated with impaired wound healing and occurrence of diabetes after transplantation (19). A systematic review and meta-analysis (19) indicated that obesity was associated with delayed graft function (relative risk, 1.41) and death by cardiovascular disease (relative risk, 2.07) particularly in patients who received a kidney allograft before 2000 (19). Usually, a body mass index higher than 35 kg/m^2^ has been reported as a threshold for kidney transplant waiting-list access (20). There are no randomized trials available examining whether weight loss may be beneficial in these patients. Irish et al. analyzed 24,337 deceased donor renal transplant recipients who received a kidney allograft from 2003 to 2006 (21). They observed that body mass index, donation after cardiac death, donor age, donor creatinine, and cold ischemia time predicted acute allograft injury (21). Molnar et al. investigated the association between pretransplant blood pressure in 13,881 hemodialysis patients and posttransplant mortality (22). Recipients with post-dialysis diastolic blood pressure more than 100 mmHg had a significantly higher risk of death (HR, 3.50) compared to patients with post-dialysis diastolic blood pressure of 70 to 80 mmHg (22).

Comorbidities including diabetes, several types of cardiovascular diseases, and lung or gastrointestinal diseases in potential kidney transplant recipients may affect survival after transplantation (23). During the follow-up period of 4.6 years, Tsarpali et al. observed 66 deaths in 192 kidney transplant recipients with a mean age of 72 years. They reported that an increased Liu comorbidity index consistently predicted poor recipients’ survival (23). Park et al. reported that comorbidities including diabetes and several types of cardiovascular diseases stratified the risk for mortality in 3,765 kidney allograft recipients (24). In contrast, Schold et al. reported that clinical features outlined above may show only poor risk prediction for recipients and allograft longevity, because using models based on large data from national registries had a 1-year C-statistic of only 0.67 (25).

The Organ Procurement and Transplantation Network (OPTN) uses an Estimated Post Transplant Survival (EPTS) score (26) which is calculated from four parameters, i.e., potential kidney recipient age, time on dialysis, diabetes, and prior solid organ transplants (26). Potential kidney recipients who have a lower EPTS score will experience more years of graft function from high-longevity kidneys compared to those who have higher EPTS scores (26). Therefore, potential kidney recipients with EPTS scores lower than 20% will receive increased priority for offers for donor kidneys with Kidney Donor Profile Index (KDPI) scores lower than 20% (26).

An analysis from the U.S. Renal Data System (USRDS) registry showed that longer pretransplant time on dialysis predicted posttransplant graft loss (27). In contrast, a retrospective study by Haller et al. (28) did not confirm that longer pretransplant time on dialysis predicted graft loss, but longer pretransplant time on dialysis was associated with increased mortality (28).

## Pretransplant biomarkers and posttransplant acute allograft injury

A recent study with 806 incident kidney transplant recipients pointed to the importance of pretransplant plasma endotrophin for posttransplant allograft injury (29). Endotrophin is a proteolytic fragment of collagen type VI, generated by the cleavage from the α3 chain during protein assembly and maturation (30–32). Both studies in patients with chronic kidney disease as well as in patients with type 2 diabetes and microalbuminuria showed that higher endotrophin was associated with higher mortality (30–32). Tepel et al. showed the association of elevated pretransplant plasma endotrophin in kidney transplant recipients and elevated acute allograft injury after transplantation (29). This association could be observed in three European cohorts even after adjustment for known causes of delayed graft function (29). They reported a cutoff level of 61.65 ng/ml for pretransplant plasma endotrophin which had a negative predictive value of 0.92 (29). The odds ratio for acute allograft injury was 2.09 with a 95% confidence interval ranging from 1.30 to 3.36 (29). Pretransplant plasma endotrophin was an independent marker for acute allograft injury in both living and deceased donor transplants (29).

Pontrelli et al. showed that pretransplant transcripts of C–C chemokine receptor type 2 were higher in circulating mononuclear cells from 25 kidney transplant recipients with acute allograft injury compared to 25 controls, showing a sensitivity of 0.74 and a specificity of 0.68 (33). C–C chemokine receptor type 2, which is also called CD192, is the receptor for monocyte chemoattractant protein-1 which is involved in monocyte chemotaxis after tissue damage (33). If confirmed in future studies, potentially modifiable pretransplant biomarkers may help for kidney transplant allocation to reduce postoperative acute allograft injury.

## Pretransplant biomarkers and posttransplant rejection

Human leukocyte antigen (HLA) typing is recommended prior to transplantation, because of the known long-term influence of HLA matching on allograft survival (34). In order to identify potential kidney transplant recipients at risk for rapid allograft rejection, recipient serum is routinely screened for preexisting anti-donor HLA antibodies with either a complement-dependent cytotoxicity (CDC)-crossmatch or a flow-cytometry-crossmatch testing (35, 36). A panel reactive antibody assay and determination of donor-specific antibodies to detect for previous sensitization is recommended because donor-specific antibodies beyond a threshold of approximately 3,000 MFI units cause a 100-fold higher risk of acute humoral rejection (37). Duquesnoy et al. reported an algorithm for matching amino acid triplets between recipient and donor HLA (38). They reported that development of posttransplant donor-specific antibodies may be more likely with increased mismatches of HLA polymorphisms between recipients and donors (38).

The importance of HLA-DQ matching for transplant outcome has been recognized in recent years (39–42). Leeaphorn et al. retrospectively analyzed data of 93,782 kidney transplant recipients from the United Network for Organ Sharing (UNOS) (41). They reported that both living kidney donor recipients (hazard ratio, 1.18) as well as deceased kidney donor recipients with cold ischemic time less than 17 h (hazard ratio, 1.12) who had HLA-DQ mismatching showed a higher risk of graft loss (41). They found that kidney transplant recipients with HLA-DQ mismatches had more episodes of acute graft reception withing the first year (odds ratio, 1.13) (41). The effects of HLA-DQ mismatches may be explained in part by *de novo* anti-DQ donor-specific antibodies, which may cause acute rejection and graft glomerulopathy (39). Therefore, the question arose, whether additional matchings will be needed in kidney transplant recipients (42).

Zhang et al. investigated 235 kidney transplant recipients with pretransplant RNA sequencing (43). They showed that the reduction of gene signatures from natural killer and CD8+ T cells was associated with early acute cellular rejection (43). A 23-gene set predicted early acute cellular rejection with an area under the curve of 0.80 (43).

Using a B-cell interferon-gamma ELISPOT assay, donor-specific memory B cells can be detected pretransplant (44). Hricik et al. did not observe an association of acute rejection nor kidney function with pretransplant interferon-gamma ELISPOT positivity in 176 kidney transplant recipients (45). However, they observed that association solely in patients who did not receive induction therapy with anti-thymocyte globulin (45).

The role of soluble 120-kDa transmembrane glycoprotein (sCD30) remains unclear. A meta-analysis including 12 studies and 2,507 patients documented a poor accuracy of pretransplant sCD30 and acute rejection (46). The meta-analysis indicated that the pooled sensitivity was 0.70, whereas the specificity was 0.48 (46).

The pretransplant determination of HLA mismatches between recipients and donors is known for a long time in order to reduce allograft rejections. In addition, several non-HLA antibodies have been reported to cause rejection and allograft nephropathy (47–51). In their retrospective investigation, Lamarthée et al. reported that higher levels of non-HLA antibodies directed against endothelial cells predicted antibody-mediated rejection histology (50). Several assays have been described to detect non-HLA antibodies (51). The importance of the genetic mismatch of non-HLA haplotypes for transplant outcome had been investigated by Reindl-Schwaighofer et al. (52). They investigated the number of non-synonymous single-nucleotide polymorphism mismatches in 477 pairs of deceased donors and first kidney transplant recipients (52). They found a median of 1,892 (interquartile range, 1,850–1,936) non-synonymous single-nucleotide polymorphism mismatches between donors and recipients (52). Elevated numbers of non-synonymous single-nucleotide polymorphism mismatch were associated with increased graft loss in a multivariable analysis (52).

In summary, determination of anti-HLA antibodies, HLA-DQ mismatches, and non-HLA antibodies are and/or will be important to reduce rejection episodes after kidney transplantation.

## Pretransplant biomarkers and long-term outcome

Pretransplant serum NT-proBNP, a marker associated with volume overload and congestive heart failure, was independently associated with all-cause mortality in 658 kidney transplant recipients during a median follow-up of 12.7 years (53). The hazard rate per 1 standard deviation of NT-proBNP increase was 1.67 with a 95% confidence interval of 1.48 to 1.89 (53). Even after adjusting for age, gender, and several other factors, the association between NT-proBNP and mortality remained significant (53).

In their retrospective study, Gomez et al. found that an elevated pretransplant serum growth differentiation factor of 15 in 359 kidney transplant recipients was associated with posttransplant mortality (hazard rate, 2.2) during a follow-up of 15 years (54). Growth differentiation factor 15 is expressed in several organs and may be upregulated after tissue damage. The authors concluded that growth differentiation factor 15 may be superior to troponin I to predict cardiovascular events after kidney transplant (54).

Some markers including kidney injury molecule-1, neutrophil gelatinase-associated lipocalin, calprotectin, and clusterin have been investigated posttransplant to indicate acute allograft injury (55–57). The levels of these early markers are rising only after acute allograft injury has occurred. For example, higher posttransplant urinary calprotectin levels predicted a lower glomerular filtration rate 12 months after transplantation (57). Another example, elevated levels of urinary clusterin 4 h after transplant could be observed in recipients with acute kidney injury (58). However, it is unknown whether the determination of these markers pretransplant may also help to evaluate posttransplant outcome. A study with a follow-up period of 12 months in 135 solid organ transplant recipients indicated that pretransplant cytomegalovirus-specific T-cell immunity (CD8+CD69+INF-γ+ T cells greater than 0.25%) was associated with lower cytomegalovirus infections posttransplant (59).

**Figure 1:**
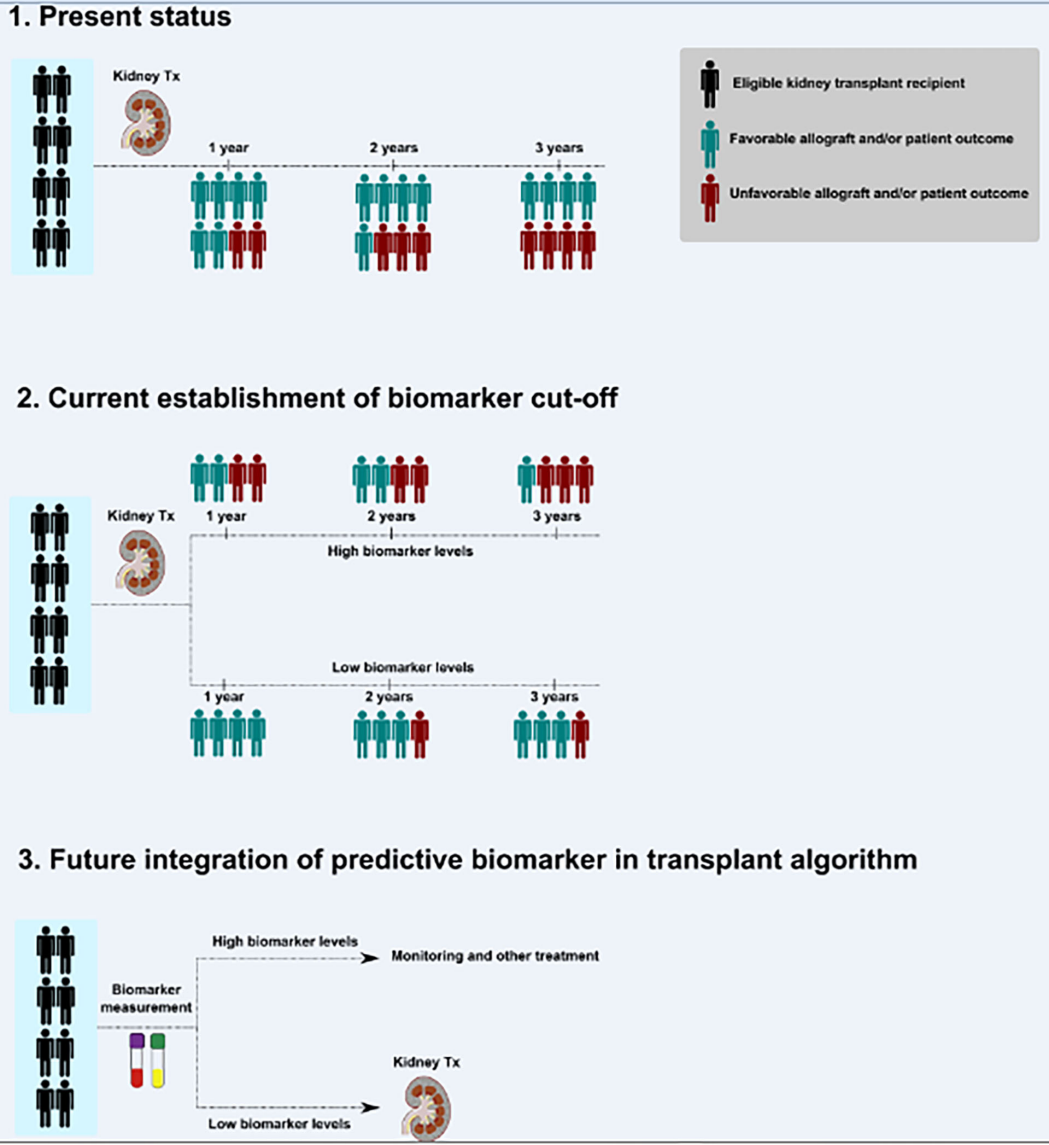
Steps toward the future integration of a predictive biomarker to evaluate potential kidney transplant recipients. A simplified scheme for investigations required to improve future transplant medicine and to reach precision medicine is shown. Outcome may indicate a large spectrum of paraclinical or clinical endpoints, for example (but not limited to), glomerular filtration rate, loss of allograft, infections, hypertension, immunosuppression, acute allograft injury, rejection episodes, or patient-reported outcome measures. Examples for outcome variables’ timing (years) are shown but may be adopted according to appropriate study plans. Tx indicates transplantation.

The steps toward future integration of the predictive biomarker, for example endotrophin, to evaluate potential kidney transplant recipients are summarized in **Figure 1**. The predictive biomarker will indicate which therapeutic option (transplantation vs. monitoring and other treatment) would be reasonable for the potential kidney transplant recipient.

## Conclusion

It is concluded that there is a need for integration of clinical characteristics and immunological and molecular biomarkers to improve future transplant medicine to reach better diagnostic decisions tailored to the individual patient.

## Author contributions

MT prepared the first outline and integrated and edited contributions from all coauthors. MT, SN, QS OT, SJLB, JB, MAK, FG, and DGKR have contributed significantly to the text of the review, discussed the overall concept, contributed to specific sections, and reviewed the manuscript. MT, SN, QS, FG, and DGKR analyzed the current literature and have written main parts of the text and figure. OT, SJB, JB, and MAK revised the text and the figure. All authors approved the final version and submission of the manuscript.

## Funding

The funder had no role in the writing of the manuscript or the decision to submit it for publication. The funder had no role in data collection, analysis, or interpretation; trial design; patient recruitment; or any aspect pertinent to the study. There was no payment for writing this article by a pharmaceutical company or other agency. This study was supported by a grant from the European Union, Eurostars (Project E!12850 PRO-C6-Rec), Innovation Fund Denmark (Eurostars Project E!12850 PRO-C6-Rec and 9046-00025B, 2019), and the Danish Research Foundation.

## Conflict of interest

MAK, FG, and DGKR are full-time employees at Nordic Bioscience. Nordic Bioscience is a privately-owned, small–medium size enterprise partly focused on the development of biomarkers. None of the authors received fees, bonuses or other benefits for the work described in the review. MAK, FG, and DGKR hold stocks in Nordic Bioscience.

The remaining authors declare that the research was conducted in the absence of any commercial or financial relationships that could be construed as a potential conflict of interest.

## Publisher’s note

All claims expressed in this article are solely those of the authors and do not necessarily represent those of their affiliated organizations, or those of the publisher, the editors and the reviewers. Any product that may be evaluated in this article, or claim that may be made by its manufacturer, is not guaranteed or endorsed by the publisher.

## References

[1] Danovitch GM ed. Handbook of kidney transplantation. 4th ed. Philadelphia: Lippincott, Williams & Wilkins (2005).

[2] Wu WK Famure O Li Y Kim SJ. Delayed graft function and the risk of acute rejection in the modern era of kidney transplantation. Kidney Int (2015) 88:851–8. doi: 10.1038/ki.2015.190 26108067

[3] El-Husseini A Aghil A Ramirez J Sawaya B Rajagopalan N Baz M. Outcome of kidney transplant in primary, repeat, and kidney-after-nonrenal solid-organ transplantation: 15-year analysis of recent UNOS database. Clin Transplant (2017) 31. doi: 10.1111/ctr.13108 28881060

[4] Raynaud M Aubert O Divard G Reese PP Kamar J Yoo D. Dynamic prediction of renal survival among deeply phenotyped kidney transplant recipients using artificial intelligence: an observational, international, multicohort study. Lancet Digit Health (2021) 3:e795–805. doi: 10.1016/S2589-7500(21)00209-0 34756569

[5] Kaplan B Schold J Meier-Kriesche HU. Poor predictive value of serum creatinine for renal allograft loss. Am J Transplant (2003) 3:1560–5. doi: 10.1046/j.1600-6135.2003.00275.x 14629286

[6] He X Moore J Shabir S Little MA Cockwell P Ball S. Comparison of the predictive performance of eGFR formulae for mortality and graft failure in renal transplant recipients. Transplantation (2009) 87:384–92. doi: 10.1097/TP.0b013e31819004a1 19202443

[7] Naesens M Lerut E Emonds MP Herelixka H Evenepoel P Claes K. Proteinuria as a noninvasive marker for renal allograft histology and failure: An observational cohort study. J Am Soc Nephrol (2016) 27:281–92. doi: 10.1681/ASN.2015010062 PMC4696583 26152270

[8] Foucher Y Daguin P Akl A Kessler M Ladrière M Legendre C. A clinical scoring system highly predictive of long-term kidney graft survival. Kidney Int (2010) 78:1288–94. doi: 10.1038/ki.2010.232 20861817

[9] Loupy A Aubert O Orandi BJ Naesens M Bouatou Y Raynaud M. Prediction system for risk of allograft loss in patients receiving kidney transplants: international derivation and validation study. BMJ (2019) 366:l4923. doi: 10.1136/bmj.l4923 31530561 PMC6746192

[10] Bontha SV Maluf DG Mueller TF Mas VR. Systems biology in kidney transplantation: The application of multi-omics to a complex model. Am J Transplant (2017) 17:11–21. doi: 10.1111/ajt.13881 27214826

[11] Ho J Rush DN Nickerson PW. Urinary biomarkers of renal transplant outcome. Curr Opin Organ Transplant (2015) 20:476–81. doi: 10.1097/MOT.0000000000000208 26107968

[12] Menon MC Murphy B Heeger PS. Moving biomarkers toward clinical implementation in kidney transplantation. J Am Soc Nephrol (2017) 28:735–47. doi: 10.1681/ASN.2016080858 PMC5328171 28062570

[13] Naesens M Anglicheau D. Precision transplant medicine: Biomarkers to the rescue. J Am Soc Nephrol (2018) 29:24–34. doi: 10.1681/ASN.2017010004 28993504 PMC5748900

[14] Stewart DE Kucheryavaya AY Klassen DK Turgeon NA Formica RN Aeder MI. Changes in deceased donor kidney transplantation one year after KAS implementation. Am J Transplant (2016) 16:1834–47. doi: 10.1111/ajt.13770 26932731

[15] Williams RC Opelz G McGarvey CJ Weil EJ Chakkera HA. The risk of transplant failure with HLA mismatch in first adult kidney allografts from deceased donors. Transplantation (2016) 100:1094–102. doi: 10.1097/TP.0000000000001115 PMC8086563 26901078

[16] Neri F Furian L Cavallin F Ravaioli M Silvestre C Donato P. How does age affect the outcome of kidney transplantation in elderly recipients? Clin Transplant (2017) 31. doi: 10.1111/ctr.13036 28640530

[17] Balzer MS Pankow S Claus R Dumann E Ruben S Haller H. Pretransplant dialysis modality and long-term patient and kidney allograft outcome: a 15-year retrospective single-centre cohort study. Transpl Int (2020) 33:376–90. doi: 10.1111/tri.13552 31705694

[18] Lin HT Liu FC Lin JR Pang ST Yu HP. Impact of the pretransplant dialysis modality on kidney transplantation outcomes: a nationwide cohort study. BMJ Open (2018) 8:e020558. doi: 10.1136/bmjopen-2017-020558 PMC5988177 29866727

[19] Nicoletto BB Fonseca NK Manfro RC Gonçalves LF Leitão CB Souza GC. Effects of obesity on kidney transplantation outcomes: a systematic review and meta-analysis. Transplantation (2014) 98:167–76. doi: 10.1097/TP.0000000000000028 24911038

[20] Dziodzio T Hillebrandt KH Knitter S Nösser M Globke B Ritschl PV Biebl M. German Bariatric surgery, kidney transplantation group. body mass index thresholds and the use of bariatric surgery in the field of kidney transplantation in Germany. Obes Surg (2022) 32:1641–8. doi: 10.1007/s11695-022-06000-4 PMC8986752 35305229

[21] Irish WD Ilsley JN Schnitzler MA Feng S Brennan DC. A risk prediction model for delayed graft function in the current era of deceased donor renal transplantation. Am J Transplant (2010) 10:2279–86. doi: 10.1111/j.1600-6143.2010.03179.x 20883559

[22] Molnar MZ Foster CE 3rd Sim JJ Remport A Krishnan M Kovesdy CP. Association of pre-transplant blood pressure with posttransplant outcomes. Clin Transplant (2014) 28:166–76. doi: 10.1111/ctr.12292 PMC3946323 24372673

[23] Tsarpali V Midtvedt K Lønning K Bernklev T Åsberg A Fawad H. A comorbidity index and pretransplant physical status predict survival in older kidney transplant recipients: A national prospective study. Transplant Direct (2022) 8:e1307. doi: 10.1097/TXD.0000000000001307 35350108 PMC8947685

[24] Park JY Kim MH Bae EJ Kim S Kim DK Joo KW. Comorbidities can predict mortality of kidney transplant recipients: Comparison with the charlson comorbidity index. 10.1016/j.transproceed.2018.01.044 29731067

[25] Schold JD Srinivas TR Howard RJ Jamieson IR Meier-Kriesche HU. The association of candidate mortality rates with kidney transplant outcomes and center performance evaluations. Transplantation (2008) 85:1–6. doi: 10.1097/01.tp.0000297372.51408.c2 18192903

[26] Organ Procurement and Transplantation Network (OPTN). Estimated post transplant survival (EPTS) score . Available at: https://optn.transplant.hrsa.gov/resources/allocation-calculators/epts-calculator/ https://optn.transplant.hrsa.gov/media/1511/guide_to_calculating_interpreting_epts.pdf.

[27] Meier-Kriesche HU Schold JD. The impact of pretransplant dialysis on outcomes in renal transplantation. Semin Dial (2005) 18:499–504. doi: 10.1111/j.1525-139X.2005.00096.x 16398713

[28] Haller MC Kainz A Baer H Oberbauer R. Dialysis vintage and outcomes after kidney transplantation: A retrospective cohort study. Clin J Am Soc Nephrol (2017) 12:122–30. doi: 10.2215/CJN.04120416 PMC5220655 27895135

[29] Tepel M Alkaff FF Kremer D Bakker SJL Thaunat O Nagarajah S. Pretransplant endotrophin predicts delayed graft function after kidney transplantation. Sci Rep (2022) 12:4079. doi: 10.1038/s41598-022-07645-y 35260630 PMC8904626

[30] Fenton A Jesky MD Ferro CJ Sørensen J Karsdal MA Cockwell P. Serum endotrophin, a type VI collagen cleavage product, is associated with increased mortality in chronic kidney disease. PloS One (2017) 12(4):e0175200. doi: 10.1371/journal.pone.0175200 28403201 PMC5389629

[31] Rasmussen DGK Fenton A Jesky M Ferro C Boor P Tepel M. Urinary endotrophin predicts disease progression in patients with chronic kidney disease. Sci Rep (2019) 7:17328. doi: 10.1038/s41598-017-17470-3 PMC5725589 29229941

[32] Rasmussen DGK Hansen TW von Scholten BJ Nielsen SH Reinhard H Parving HH. Higher collagen VI formation is associated with all-cause mortality in patients with type 2 diabetes and microalbuminuria. Diabetes Care (2018) 41:1493–500. doi: 10.2337/dc17-2392 29643059

[33] Pontrelli P Simone S Rascio F Pesce F Conserva F Infante B. Pre-transplant expression of CCR-2 in kidney transplant recipients is associated with the development of delayed graft function. Front Immunol (2022) 13:804762. doi: 10.3389/fimmu.2022.804762 35371047 PMC8967482

[34] Süsal C Opelz G. Current role of human leukocyte antigen in kidney transplantation. Curr Opin Organ Transplant (2013) 18:438. doi: 10.1097/MOT.0b013e3283636ddf 23838649

[35] Terasaki PI Ozawa M. Predicting kidney graft failure by HLA antibodies: a prospective trial. Am J Transplant (2004) 4:438–43. doi: 10.1111/j.1600-6143.2004.00360.x 14961999

[36] Reed EF Rao P Zhang Z Gebel H Bray RA Guleria I. GjertsonD: Comprehensive assessment and standardization of solid phase multiplex- bead arrays for the detection of antibodies to HLA-drilling down on key sources of variation. Am J Transplant (2013) 13:3050–1. doi: 10.1111/ajt.12462 PMC3837625 24103158

[37] Lefaucheur C Loupy A Hill GS Andrade J Nochy D Antoine C. Preexisting donor-specific HLA antibodies predict outcome in kidney transplantation. J Am Soc Nephrol (2010) 21:1398–406. doi: 10.1681/ASN.2009101065 PMC2938596 20634297

[38] Duquesnoy RJ Takemoto S de Lange P Doxiadis II Schreuder GM Persijn GG. HLAMatchmaker. a molecularly based algorithm for histocompatibility determination. III. effect of matching at the HLA-A,B amino acid triplet level on kidney transplant survival. Transplantation (2003) 75:884–9. doi: 10.1097/01.TP.0000055101.20821.AC 12660519

[39] DeVos JM Gaber AO Knight RJ Land GA Suki WN Gaber LW. Donor-specific HLA-DQ antibodies may contribute to poor graft outcome after renal transplantation. Kidney Int (2012) 82:598–604. doi: 10.1038/ki.2012.190 22622504

[40] Lim WH Chapman JR Coates PT Lewis JR Russ GR Watson N. HLA-DQ mismatches and rejection in kidney transplant recipients. Clin J Am Soc Nephrol (2016) 11:875–83. doi: 10.2215/CJN.11641115 PMC4858494 27034399

[41] Leeaphorn N Pena JRA Thamcharoen N Khankin EV Pavlakis M Cardarelli F. HLA-DQ mismatching and kidney transplant outcomes. Clin J Am Soc Nephrol (2018) 13:763–71. doi: 10.2215/CJN.10860917 PMC5968890 29685925

[42] Süsal C Zeier M. Is there a need for additional DQ matching? Clin J Am Soc Nephrol (2018) 13:683–4. doi: 10.2215/CJN.03720318 PMC5969472 29685926

[43] Zhang W Yi Z Wei C Keung KL Sun Z Xi C. Pretransplant transcriptomic signature in peripheral blood predicts early acute rejection. JCI Insight (2019) 4:e127543. doi: 10.1172/jci.insight.127543 PMC6629121 31167967

[44] Czerkinsky CC Nilsson LA Nygren H Ouchterlony O Tarkowski A. A solid-phase enzyme-linked immunospot (ELISPOT) assay for enumeration of specific antibody secreting cells. J Immunol Methods (1983) 65:109–21. doi: 10.1016/0022-1759(83)90308-3 6361139

[45] Hricik DE Augustine J Nickerson P Formica RN Poggio ED Rush D. Interferon gamma ELISPOT testing as a risk-stratifying biomarker for kidney transplant injury: Results from the CTOT-01 multicenter study. Am J Transplant (2015) 15:3166–73. doi: 10.1111/ajt.13401 PMC4946339 26226830

[46] Chen Y Tai Q Hong S Kong Y Shang Y Liang W. Pretransplantation soluble CD30 level as a predictor of acute rejection in kidney transplantation: a meta-analysis. Transplantation (2012) 94:911–8. doi: 10.1097/TP.0b013e31826784ad 23052636

[47] Dragun D Müller DN Bräsen JH Fritsche L Nieminen-Kelhä M Dechend R. Angiotensin II type 1-receptor activating antibodies in renal-allograft rejection. N Engl J Med (2005) 352:558–69. doi: 10.1056/NEJMoa035717 15703421

[48] Zou Y Stastny P Süsal C Döhler B Opelz G. Antibodies against MICA antigens and kidney-transplant rejection. N Engl J Med (2007) 357:1293–300. doi: 10.1056/NEJMoa067160 17898098

[49] Jethwani P Rao A Bow L Menon AC. Donor-recipient non-HLA variants, mismatches and renal allograft outcomes: Evolving paradigms. Front Immunol (2022) 13:822353. doi: 10.3389/fimmu.2022.822353 35432337 PMC9012490

[50] Lamarthée B Burger C Leclaire C Lebraud E Zablocki A Morin L. CRISPR/Cas9-engineered HLA-deleted glomerular endothelial cells as a tool to predict pathogenic non-HLA antibodies in kidney transplant recipients. J Am Soc Nephrol (2021) 32:3231–51. doi: 10.1681/ASN.2021050689 PMC8638404 35167486

[51] Lammerts RGM Altulea D Hepkema BG Sanders JS van den Born J Berger SP. Antigen and cell-based assays for the detection of non-HLA antibodies. Front Immunol (2022) 13:864671. doi: 10.3389/fimmu.2022.864671 35603145 PMC9122123

[52] Reindl-Schwaighofer R Heinzel A Kainz A van Setten J Jelencsics K Hu K. Contribution of non-HLA incompatibility between donor and recipient to kidney allograft survival: genome-wide analysis in a prospective cohort. Lancet (2019) 393:910–7. doi: 10.1016/S0140-6736(18)32473-5 30773281

[53] Yeung SMH van Londen M Nakshbandi U Said MY Eisenga MF Hepkema BG. Pretransplant NT-proBNP, dialysis vintage, and posttransplant mortality in kidney transplant recipients. Transplantation (2020) 104:2158–65. doi: 10.1097/TP.0000000000003125 31978004

[54] Gomez M Unzueta MTG Hernandez AB Fernandez AA Arnedo MP Cuesta CL. Growth differentiation factor 15 is superior to troponin I in the evaluation of kidney transplant candidates. Am J Nephrol (2022) 53:118–28. doi: 10.1159/000521781 35196660

[55] van Timmeren MM Vaidya VS van Ree RM Oterdoom LH de Vries APJ Gans ROB. High urinary excretion of kidney injury molecule-1 is an independent predictor of graft loss in renal transplant recipients. Transplantation (2007) 84:1625–30. doi: 10.1097/01.tp.0000295982.78039.ef PMC2745062 18165774

[56] Hall IE Yarlagadda SG Coca SG Wang Z Doshi M Devarajan P. IL-18 and urinary NGAL predict dialysis and graft recovery after kidney transplantation. J Am Soc Nephrol (2010) 21:189–97. doi: 10.1681/ASN.2009030264 PMC2799276 19762491

[57] Tepel M Borst C Bistrup C Marcussen N Pagonas N Seibert FS. Urinary calprotectin and posttransplant renal allograft injury. PloS One (2014) 9:e113006. doi: 10.1371/journal.pone.0113006 25402277 PMC4234472

[58] Pianta TJ Peake PW Pickering JW Kelleher M Buckley NA Endre ZH. Clusterin in kidney transplantation: novel biomarkers versus serum creatinine for early prediction of delayed graft function. Transplantation (2015) 99:171–9. doi: 10.1097/TP.0000000000000256 25083615

[59] Molina-Ortega A Martín-Gandul C Mena-Romo JD Rodríguez-Hernández MJ Suñer M Bernal C. Impact of pretransplant CMV-specific T-cell immune response in the control of CMV infection after solid organ transplantation: a prospective cohort study. Clin Microbiol Infect (2019) 25:753–8. doi: 10.1016/j.cmi.2018.09.019 30292792

